# Integrated Modeling of Gene Regulatory and Metabolic Networks in *Mycobacterium tuberculosis*

**DOI:** 10.1371/journal.pcbi.1004543

**Published:** 2015-11-30

**Authors:** Shuyi Ma, Kyle J. Minch, Tige R. Rustad, Samuel Hobbs, Suk-Lin Zhou, David R. Sherman, Nathan D. Price

**Affiliations:** 1 Department of Chemical and Biomolecular Engineering, University of Illinois, Urbana-Champaign, Illinois, United States of America; 2 Institute for Systems Biology, Seattle, Washington, United States of America; 3 Center for Infectious Disease Research, formerly Seattle Biomedical Research Institute, Seattle, Washington, United States of America; 4 Interdisciplinary Program of Pathobiology, Department of Global Health, University of Washington, Seattle, Washington, United States of America; ETH Zurich, SWITZERLAND

## Abstract

*Mycobacterium tuberculosis* (MTB) is the causative bacterium of tuberculosis, a disease responsible for over a million deaths worldwide annually with a growing number of strains resistant to antibiotics. The development of better therapeutics would greatly benefit from improved understanding of the mechanisms associated with MTB responses to different genetic and environmental perturbations. Therefore, we expanded a genome-scale regulatory-metabolic model for MTB using the Probabilistic Regulation of Metabolism (PROM) framework. Our model, *MTB*PROM2.0, represents a substantial knowledge base update and extension of simulation capability. We incorporated a recent ChIP-seq based binding network of 2555 interactions linking to 104 transcription factors (TFs) (representing a 3.5-fold expansion of TF coverage). We integrated this expanded regulatory network with a refined genome-scale metabolic model that can correctly predict growth viability over 69 source metabolite conditions and predict metabolic gene essentiality more accurately than the original model. We used *MTB*PROM2.0 to simulate the metabolic consequences of knocking out and overexpressing each of the 104 TFs in the model. *MTB*PROM2.0 improves performance of knockout growth defect predictions compared to the original PROM MTB model, and it can successfully predict growth defects associated with TF overexpression. Moreover, condition-specific models of *MTB*PROM2.0 successfully predicted synergistic growth consequences of overexpressing the TF *whiB4* in the presence of two standard anti-TB drugs. *MTB*PROM2.0 can screen *in silico* condition-specific transcription factor perturbations to generate putative targets of interest that can help prioritize future experiments for therapeutic development efforts.

## Author Summary

Tuberculosis remains a major global health challenge with a need for enhanced drug development efforts. Drug development would be aided by understanding more about the bacteria that causes the disease, *Mycobacterium tuberculosis* (MTB), and how it adapts to survive the broad range of conditions within hosts. To help this effort, we extended a computational model that uses our understanding of how the MTB transcriptional regulatory network (genes that interact to control the abundance of target genes) influences the metabolic network (genes that drive biochemical reactions). Using this model, *MTB*PROM2.0, we were able to successfully predict whether disrupting or boosting the action of regulatory genes would cause a growth defect in MTB. By applying these predictions, across many environmental conditions, this tool can help find potential new drug targets for more effective MTB treatments.

## Introduction

Tuberculosis (TB) remains a major global health challenge with a need for enhanced drug development efforts. The current standard treatment for drug-susceptible tuberculosis involves treating for 6 months with a cocktail of first-line drugs that have serious side-effects [1]. Antibiotic resistance, drug toxicity, and the long treatment duration renders it difficult to treat patients successfully [2]. Despite this, the current first-line therapy recommended by the World Health Organization is decades old [2].

*Mycobacterium tuberculosis* (MTB) has the ability to tune its physiology to adapt and survive in the broad range of conditions within the host, altering its drug susceptibility in the process. A promising approach for discovering novel drug targets has been to develop computational models that recapitulate the organismal phenotypes of interest. For example, to identify candidate metabolic drug targets, genome-scale mechanistic models of metabolism have been used to simulate growth and metabolic phenotypes under gene deletion conditions for multiple pathogens [3]. Another important biological network for which mechanistic models have been constructed is gene regulation[4, 5]. Transcriptional regulatory networks (TRNs) orchestrate organismal responses to condition-specific perturbations. Although TRNs and metabolic networks have been modeled mainly using different approaches, regulatory and metabolic responses are intrinsically interconnected. Modeling the metabolic consequences of regulatory perturbations in MTB will refine our understanding of its physiology and potentially lead to novel candidate drug targets.

Thus far, the effects of gene regulation have been integrated into constraint-based metabolic modeling with three major strategies: (1) modifying the objective function of a metabolic model to impose an implicit regulatory goal [6, 7], (2) overlaying gene expression information to impose condition-specific flux constraints on the metabolic model [7–11], and (3) integrating transcriptional regulatory information explicitly with the metabolic model [12–16]. Overlaying gene expression directly onto metabolic models has been used as a proxy for explicit reconstruction of gene regulatory networks to constrain the space of possible metabolic flux states. For example, the iMAT method uses ‘ON’ vs. ‘OFF’ gene expression states to guide which corresponding metabolic reactions are active vs. inactive [9, 10]. As this approach applies the effects of perturbations apparent in the gene expression data directly as reaction constraints upon the metabolic model, a model representing the perturbation of a transcription factor (TF) based on directly overlaying gene expression data requires, as input, transcriptional profiles measuring the perturbation of the specific TF of interest.

In the absence of such condition-specific gene expression data, efforts at integrating information about regulation and metabolism have reconstructed known regulatory interactions between transcription factors, metabolic target genes, and environmental conditions. These approaches do not require the generation of transcriptional profiles directly probing transcription factor perturbations, and thus are applicable to new conditions. Most of these approaches (e.g. rFBA and SR-FBA) involve manually defining a set of Boolean regulatory rules based on literature information [12–15]. A significant drawback to these manually generated rules is the difficulty to scale the rule generation process to capture the complete set of regulatory interactions for an entire organism. Regulatory constraints have also been interfaced in a semi-automated manner with the Probabilistic Regulation of Metabolism framework (PROM) [16]. PROM simulates the metabolic effect of a TF knockout by estimating from gene expression data the conditional probability that each TF knockout would disrupt the expression of a corresponding metabolic target gene, based on the following equation: $P(Gene\;=\;ON\;|\;TF\;=\;OFF)\;\approx \frac{\text{Number of samples with Gene = }ON\text{ AND TF = }OFF}{\text{Number of samples with TF = }OFF}$. This probability is mapped onto the reactions catalyzed by the protein encoded by the target gene, and it is used to modulate the maximum reaction flux bounds. Notably, the first integrated metabolic-regulatory model for MTB was constructed using PROM [16].

In this work, we present a significantly improved regulatory-metabolic model for MTB based on the PROM framework. *MTB*PROM2.0 encapsulates a substantially expanded knowledge base of underlying metabolic and regulatory mechanisms, featuring a regulatory network for 104 TFs based on ChIP-seq interactions that interface with a refined genome-scale metabolic model with 810 genes. We also extended the PROM framework to enable the prediction of metabolic consequences of TF overexpression. Using this expanded model, we can generate both knockout and overexpression phenotype predictions for 3.5 times as many TFs while exposed to a greater range of environmental and genetic perturbations. *MTB*PROM2.0 has improved agreement between predictions and experimental datasets assessing gene essentiality and overexpression growth defects compared to alternative methods, and it has successfully predicted synergy between TF perturbations and anti-TB drugs.

## Results

### Updated regulatory-metabolic model expands mechanistic knowledge base

**Fig 1:**
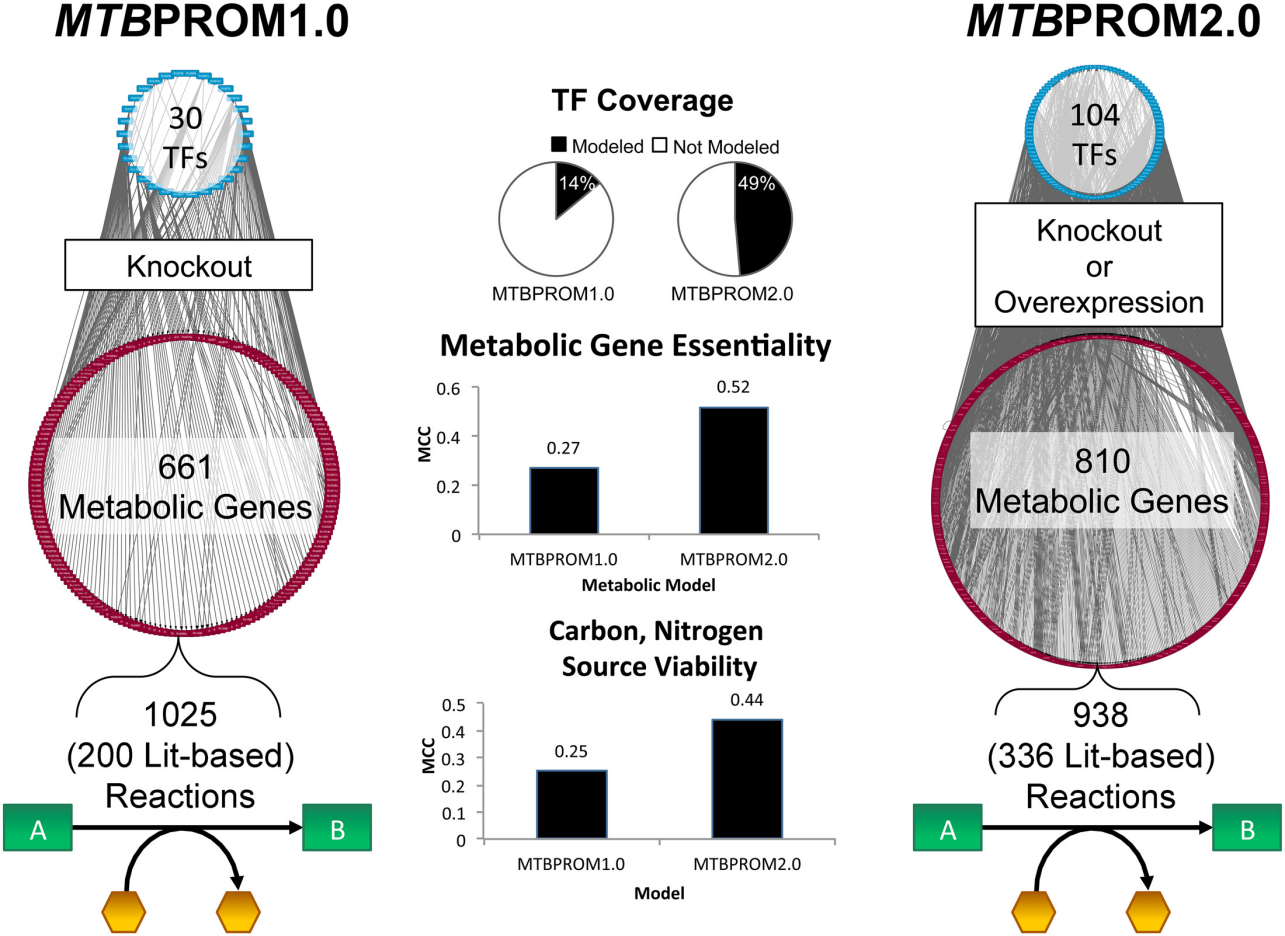
Comparison of the regulatory-metabolic model attributes. The features are compared between the initial regulatory-metabolic model constructed for MTB described in [16] (*MTB*PROM1.0) and the updated model (*MTB*PROM2.0). *MTB*PROM2.0 contains additional coverage of regulation and metabolism, with improved prediction of essential metabolic genes and growth viability in carbon and nitrogen sources, as quantified by the Matthews Correlation Coefficient (MCC, see Methods). The PROM simulation framework has also been extended to predict TF overexpression in addition to knockout phenotypes.

We have reconstructed an updated regulatory-metabolic model for MTB using the PROM framework. The updated model, *MTB*PROM2.0, incorporates a knowledge expansion in both the metabolic and regulatory components. Fig 1 summarizes the updated information incorporated into the model (additional information is provided in Table 1, S1 Text, and S1 Table).

**Table 1:** Summary of the updated integrated regulatory-metabolic model properties. The model attributes are compared between the initial regulatory-metabolic model constructed for MTB described in [16] (*MTB*PROM1.0) and the updated model (*MTB*PROM2.0). The updated model incorporates updated and significantly more data-rich representations of metabolism and gene regulation.

Feature	*MTB*PROM1.0 model	*MTB*PROM2.0 model
**Metabolic model**	iNJ661 [17]	iSM810
**Number of reactions (literature based)**	1025 (200)	938 (336)
**Number of metabolic genes**	661	810
**Regulatory network**	Balazsi 2008 [18]	Minch 2015 [19]
**Number of transcription factors**	30	104
**Number of target metabolic genes**	178	647
**Number of interactions**	218	2555

To expand the knowledge base of metabolism represented by *MTB*PROM2.0, we updated the metabolic component by integrating gene-associated reactions with literature evidence from the multiple existing genome-scale metabolic models and supplementing with additional reaction information from literature (see S1 Text for details) [20–23]. The updated metabolic model, *Mycobacterium tuberculosis* iSM810, includes a greater number of genes and reactions with literature evidence compared to the metabolic component used in *MTB*PROM1.0, which was iNJ661 (see S1 Table for the details of the properties of iSM810 and S1 File for both the SBML format and COBRA Toolbox implementation of iSM810). Moreover, iSM810 demonstrates an improved ability to predict MTB growth, as quantified by the Matthews Correlation Coefficient, which is a performance metric that reduces bias for uneven category sizes (MCC, see Methods for details). Across 91 different metabolite utilization conditions, iSM810 had an MCC = 0.44, precision = 0.71, and recall = 0.84, whereas iNJ661 had an MCC = 0.25, precision = 0.79, and recall = 0.26. For metabolic gene knockout conditions, iSM810 had an MCC = 0.52, precision = 0.83, and recall = 0.59 over 810 genes, whereas iNJ661 had an MCC = 0.27, precision = 0.72, recall = 0.44 over 661 metabolic genes (see S1 Text for detailed growth simulation results).

The transcriptional regulatory network component represents the most substantial expansion of knowledge in *MTB*PROM2.0 (see S1 Text for a visual comparison of the networks). We leveraged a greatly extended regulatory network based on transcription factor (TF) binding measured using genome-scale ChIP-seq data generated for 190 transcription factors (89% of an estimated 214 MTB TFs) (described in [19]). For the purposes of integrating with the regulatory-metabolic model, we used the subset of interactions from the ChIP-seq binding network wherein the TF binding footprint is located proximally to transcriptional start sites (i.e. region spanning 150 bp upstream to 70 bp downstream of start site), and included indirect interactions based on information from the TBDB operon browser [24, 25]. We also considered only interactions between the TFs and the genes in the metabolic model iSM810. The resulting updated regulatory component includes 2555 interactions for 104 TFs (49%) that link to 647 metabolic genes (80% of the metabolic genes in iSM810, 16% of all MTB genes). Importantly, the expanded, data-rich coverage of transcriptional regulatory interactions to metabolic genes facilitates systems modeling of a broader set of metabolic consequences to TF perturbations.

The original PROM framework was designed to simulate the metabolic effects of TF knockouts. To infer the strength of each regulatory interaction for each TF knockout simulation, the conditional probability that each target metabolic gene is expressed in the absence of each transcription factor was calculated using microarray gene expression data (see Methods for details). We used as input a dataset that measured the gene expression profiles resulting from overexpressing each of 206 TFs (which include 103 of the 104 TFs in the regulatory component of our model), as described [26]. This dataset is particularly suited to inferring the strength of each TF-target gene interaction because it measures the consequences of systematic regulatory gene perturbations. These inferred regulatory influences were mapped onto the metabolic model to simulate the consequent growth phenotype (see Methods for details).

### Updated regulatory-metabolic model improves prediction of TF gene essentiality

We used the updated regulatory-metabolic model *MTB*PROM2.0 to simulate the effect of TF knockouts on MTB growth rates across multiple media conditions (see S2 Table for complete set of simulation results). We compared the growth rate predicted for each TF knockout to the corresponding growth rate of the wild-type under the same simulated environmental conditions, and we designated TFs with simulated knockout growth rates less than 0.95 of wild-type as having growth defect.

To validate the extent to which *MTB*PROM2.0 can predict growth defects of transcription factor knockouts, we compared the simulated TF knockout predictions with experimentally derived gene essentiality data generated by Griffin et al. [27]. Our evaluation criterion was whether the model simulated knockout growth rate ratios could distinguish between the TF knockout strains that are highly confident essential (Griffin score < 0.1, 13 TFs) from TF knockouts that are highly confident non-essential (Griffin score > 0.9, 29 TFs). *MTB*PROM2.0 had an MCC = 0.33 (precision = 0.59, recall = 0.77). This improved markedly upon the overall performance of the original model, *MTB*PROM1.0, which had a MCC = 0 (precision = 0.50, recall = 0.29) on this new kind of data for comparison. *MTB*PROM2.0 performance remained higher than *MTB*PROM1.0 when other essentiality thresholds were tested as well (See S1 Text for detailed analysis).

### Updated model predicts TF overexpression growth defects

**Fig 2:**
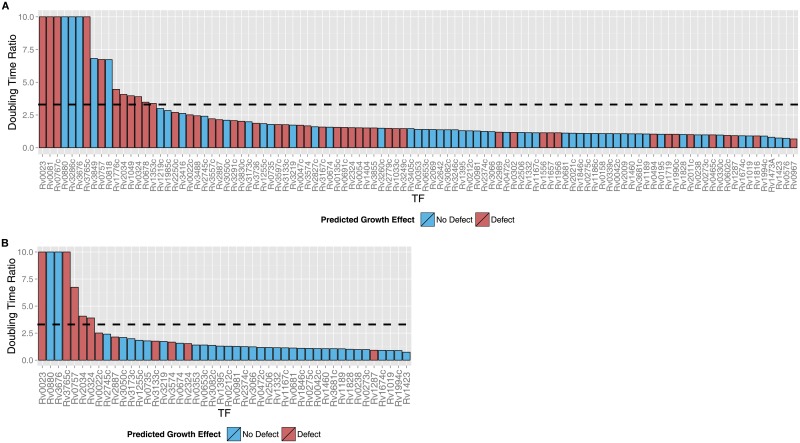
Experimental overexpressed vs. not overexpressed doubling time ratios of TFs with high confidence *MTB*PROM2.0 predictions. (A) Doubling time ratios of all TFs predicted by *MTB*PROM 2.0. (B) Doubling time ratios of the high confidence TFs identified by the logistic regression model as likely to be correctly predicted by *MTB*PROM2.0. Doubling time ratios greater than 10 are shown truncated. The bars are color-coded red if *MTB*PROM2.0 simulation predicted a growth defect upon the overexpression of each TF, and blue if no defect was predicted. The dashed line indicates the growth defect cutoff threshold at the 85^th^ percentile of doubling times.

To extend the predictive scope of the PROM framework, we modified the simulation to enable prediction of transcription factor overexpression growth phenotypes, using the same input gene expression dataset to train conditional probabilities (see Methods for details). As validation, we compared the *MTB*PROM2.0 predicted overexpression growth ratios to experimentally measured doubling time ratios of the TF overexpression strains with and without the induction of overexpression [26]. Fig 2, Panel A shows experimentally measured overexpressed vs. not overexpressed doubling time ratios of the TFs predicted by *MTB*PROM2.0, where a higher doubling time ratio indicates a greater growth defect upon TF overexpression. The bars are color-coded based on whether the *MTB*PROM2.0 simulation predicted a growth defect upon the overexpression of each TF. Using the 85th percentile (corresponding to a doubling time ratio of 3.3) as an experimental cutoff threshold to delineate growth defect vs. no defect, we evaluated the ability of MTBPROM2.0 to correctly distinguish between these groups. The overall MCC was 0.2, with precision = 0.23, and recall = 0.69.

To boost the utility of the *MTB*PROM2.0 TF overexpression predictions, we used various network properties to train a logistic regression model to estimate the confidence that each TF overexpression prediction by *MTB*PROM2.0 would be correct (see Methods for details and S3 Table for condition-specific predictions). Of the variables we tested, we found two that contributed significantly to the logistic regression model: (1) whether the *MTB*PROM2.0 prediction for a particular TF matched the prediction generated by the iMAT method [9, 10, 28], and (2) the average number of regulators that each essential target gene had (see S1 Text for complete list). We performed ten-fold cross-validation on our logistic regression model to evaluate the ability of the model to predict TFs where growth effects were correctly simulated by *MTB*PROM2.0 (see Methods and S1 Text for details), and found an average cross-validation MCC of 0.56 (precision = 0.81, recall = 0.86). This performance implies that the logistic regression model can not only be used to help prioritize the TFs that should be followed-up in further experiments, but it can also identify properties of TFs that make their phenotypes more challenging to predict by *MTB*PROM2.0.

**Fig 3:**
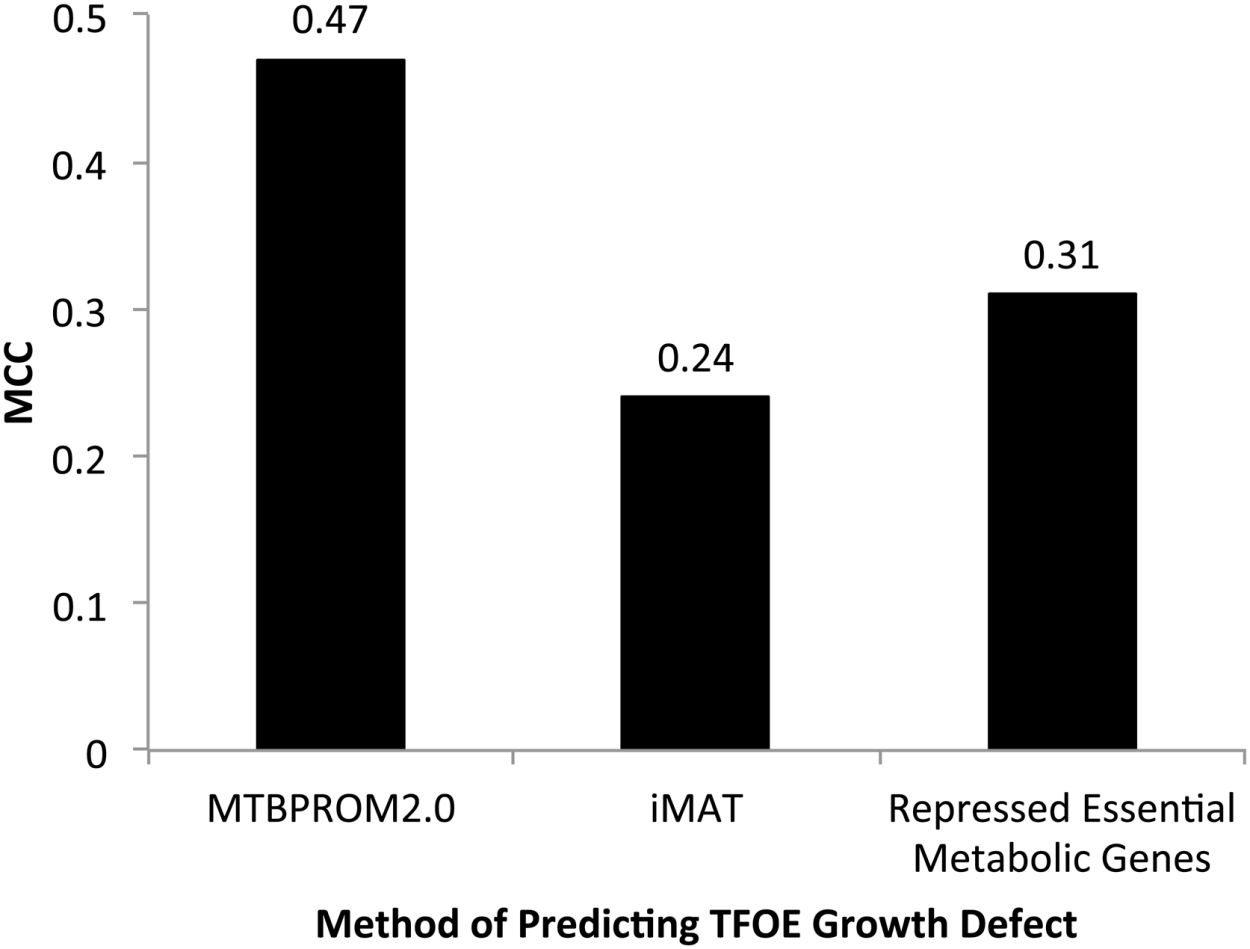
TF overexpression growth defect prediction performance. Performance of *MTB*PROM2.0 at predicting TF overexpression growth defects compared to two alternative methods: (1) iMAT and (2) whether the overexpressing TF repressed any essential metabolic genes. Performance is quantified by the MCC.

Fig 2, Panel B shows the *MTB*PROM2.0 predictions of overexpression growth defect for the 46 TFs with high confidence scores based on the logistic regression model. Using the same 85^th^ percentile cutoff threshold for growth defect as for evaluating all the TFs, *MTB*PROM2.0 predicted growth defect with a MCC of 0.47 (precision = 0.45, recall = 0.71, p < 0.01 Fisher’s exact test) (see Fig 3). *MTB*PROM2.0 achieved an improved performance compared to predicting growth defect based on the whether the TF significantly repressed essential metabolic genes (MCC = 0.31, precision = 0.26, recall = 0.88) or based on condition-specific metabolic models generated by overlaying overexpression microarray data with iMAT [9, 10, 28] (MCC = 0.24, precision = 0.31, recall = 0.50) (see Methods for details on these alternative prediction strategies).

### Predicting TF perturbations that synergize with drugs

Finding effective new drug combinations is a major challenge in the TB field. *MTB*PROM2.0 can be integrated with condition-specific metabolic models to predict effects of combinatorial perturbations on the growth of MTB. To assess our ability to predict synergies with drugs, we exploited test compounds with demonstrated anti-TB activity [29] as well as current anti-TB agents. We exposed MTB to minimum inhibitory concentration (MIC) levels of each compound for 16 hours, less than one doubling for this organism and before any cell death was evident. RNA was isolated and applied to arrays provided by TIGR under the NIAID contract N01-AI-15447 using published protocols [30] (the data are accessible from the Gene Expression Omnibus, accession number GSE71200). We used these transcriptional response data with the iMAT method to constrain iSM810 and generate a series of drug-specific models that represent the metabolic state of MTB when exposed to each of the antibacterial agents. We integrated each of these drug-specific MTB metabolic models into the updated regulatory network component of *MTB*PROM2.0 and simulated the growth outcome of each TF knockout and overexpression event. The simulations predicted multiple TF knockouts and overexpression conditions that would have a growth defect in the presence of the drug but not under standard culturing conditions (see Supplemental S1 Text and S4 Table for full results).

**Fig 4:**
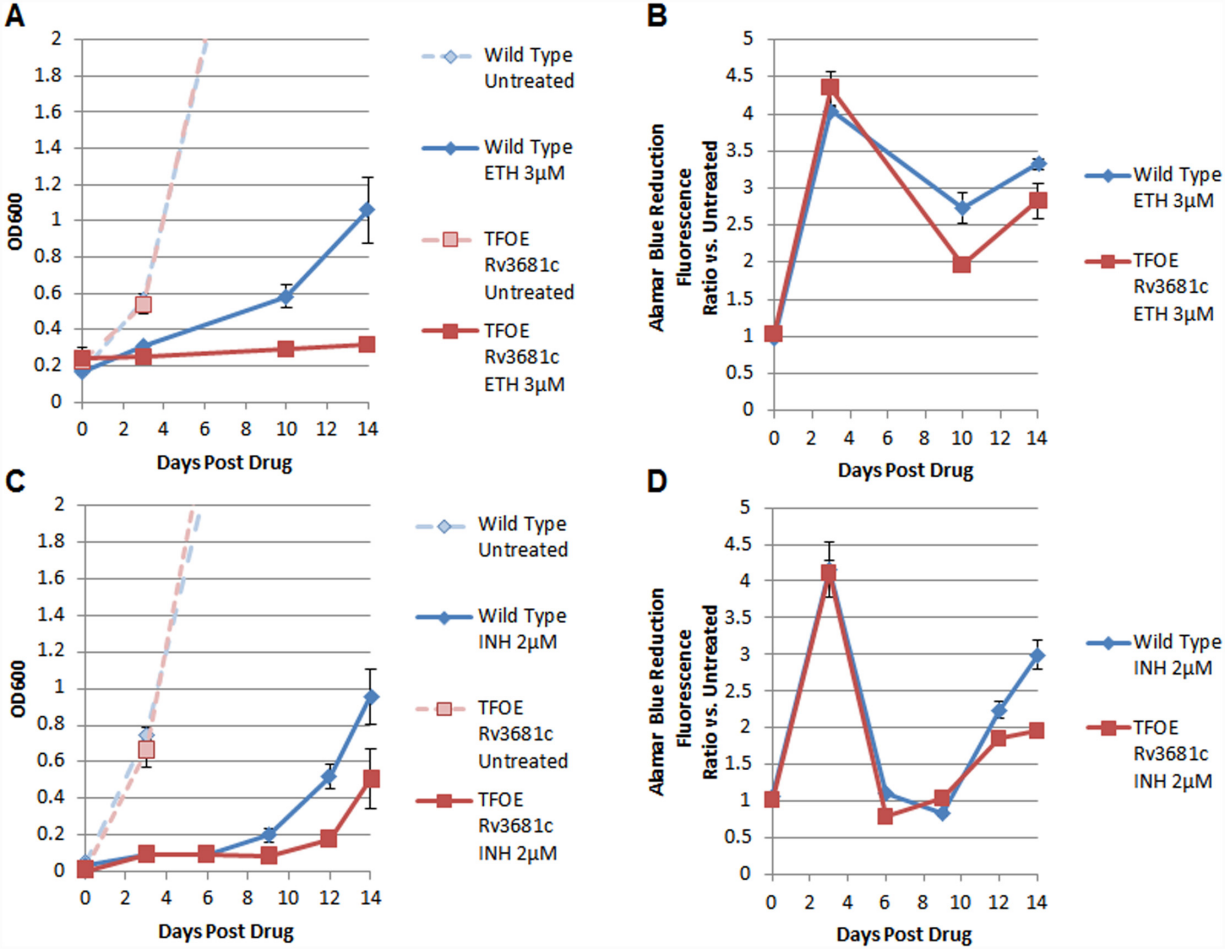
Representative time-course growth and metabolic activity of wild-type and *whiB4*-overexpression strains of MTB after treatment with drugs ethionamide (ETH) and isoniazid (INH). (A, C) The growth time-courses measured by OD600 of wild-type (blue) and *whiB4-*overexpressing MTB strains (red) without drug (pale, dashed lines) and post treatment with 3μM ETH (Panel A) and 2μM INH (Panel C). (B, D) Time-courses of metabolic activity measured by Alamar Blue reduction. Data represent mean ± standard deviation of three biological replicates.

To experimentally validate, we tested the overexpression of the TF *whiB4* (Rv3681c), which *MTB*PROM2.0 predicted to have a growth defect when exposed to each of four agents: ethionamide (ETH), isoniazid (INH), a coumarin analog (IMTB009), and a guanosine analog (IMTB0044). We compared the growth (by OD600) and metabolic activity (by Alamar Blue Assay [31]) of wild-type H37Rv with a strain overexpressing *whiB4* in the presence of each drug (see Methods for details). TF overexpression did not alter sensitivity to IMTB009 or IMTB044 (data not shown), but did synergize with the inhibitory activity of ETH and INH. Fig 4 shows representative growth and metabolic activity time-course profiles of the wild-type strain and the strain overexpressing *whiB4* from one of three experiments (each performed with three biological replicates). While no appreciable growth difference was detected between the two strains in the absence of ETH, dosing the strains with 3μM of ETH (approximately 0.5x the MIC) resulted in significantly more growth inhibition (3-fold lower OD600 at 14 days post drug, Fig 4, Panel A) and less metabolic activity (Fig 4, Panel B) in the strain overexpressing *whiB4* compared to wild-type. Similarly, dosing the strains with 2μM of INH (approximately 0.6x the MIC) resulted in significantly more growth inhibition (2-fold lower OD600 at 14 days post drug, Fig 4, Panel C) and less metabolic activity (Fig 4, Panel D) in the strain overexpressing *whiB4* compared to wild-type. In addition, we tested four drugs predicted by the model not to synergize with *whiB4* (IMTB001, IMTB031, IMTB036, and IMTB041), and observed no differential growth upon exposure to these compounds (data not shown).

## Discussion

Models that leverage knowledge of biological circuitry to generate accurate predictions of phenotype can synergize with experimental efforts to improve understanding of biology and identify novel intervention strategies. In this work, we have generated an updated mechanistic regulatory-metabolic model for *Mycobacterium tuberculosis* that incorporates expanded and improved knowledge of the metabolic and regulatory systems. The refined model, *MTB*PROM2.0, can predict metabolic consequences of TF knockout or overexpression under different environmental conditions, and suggest hypotheses of underlying molecular mechanisms that contribute to consequent phenotypes. This work has shown that we can predict growth phenotypes resulting from overexpressing or disrupting transcription factors with a mechanistic regulatory-metabolic model of MTB, and that integrating more knowledge of underlying metabolism and regulation into mechanistic models improves predictive ability. Moreover, we have demonstrated the utility of *MTB*PROM2.0 in predicting TF perturbations that synergize with the activity of drugs. Our model minimizes complicating assumptions, and with this simplified rule set, a significant proportion of predictions generated can be successfully validated. Therefore, using mechanistic models to screen and generate hypotheses for interesting targets can help to prioritize experiments that further extend knowledge of the underlying biology.

Expanding the PROM framework to enable prediction of TF overexpression phenotypes opens a new dimension of *in silico* screening that can be followed up with experiments, and to the best of our knowledge, *MTB*PROM2.0 is the first explicitly reconstructed regulatory-metabolic model to incorporate overexpression simulations. Given that the experimental tools are already in place to control and monitor the effects of TF overexpression in MTB [26], introducing this predictive capability further facilitates the capacity to translate PROM predictions into tractable experiments. Transcription factor overexpression is also a more physiological perturbation than knockout, since most transcription factors will undergo differential expression under exposure to different conditions, whereas complete gene knockout would typically arise only from genetic mutation. Being able to exploit overexpression to perturb MTB phenotype may open therapeutic possibilities for different modes of drugs. Leveraging the ability of *MTB*PROM2.0 to simulate a broader set of genetic perturbations under a greater range of conditions to guide experiments, we can begin to gain a better understanding of the condition-specific genetic sensitivities of MTB.

Incorporating additional regulatory and metabolic reaction information expanded the scope of the predictions, enabling predictions on a broader range of transcription factors than *MTB*PROM1.0. Moreover, the improvement in gene essentiality predictive performance of observed with *MTB*PROM2.0 compared to *MTB*PROM1.0 suggests that additional representation of biochemical and regulatory knowledge captured by the integrated model further drives improvements to predictive ability. Continuing efforts to improve the representation of biochemistry and regulation in the metabolic and regulatory model components—as well as inclusion of other important processes—will likely generate more comprehensive predictions that are more reflective of MTB behavior.

Finding effective combinatorial therapies is an important challenge in TB drug development. While drug combinations can be more effective and less likely to promote MTB resistance, identifying viable combinations by experimental efforts alone is hindered by the large search space. Integrating *MTB*PROM2.0 with drug-specific metabolic models informed by transcriptional responses in MTB, we successfully predicted synergistic interactions between overexpression of the transcription factor *whiB4* and the drugs ethionamide and isoniazid. This synergy suggests that the regulatory targets of *whiB4* include potential drug targets that can enhance the activity of these agents. While two other predicted *whiB4*-drug interactions were not validated, the ability of the model to identify synergistic TF-drug interactions in 50% of cases and to identify correctly drugs that do not synergize with the TF in four of four instances that we tested argues that the model is capturing a significant portion of the mechanism(s) that underlie the synergistic phenotypes. Applying this approach to other anti-TB agents could contribute to rationally informing identification of novel candidates for synergistic targets.

There remain substantial gaps in the knowledge and representation of regulation and metabolism that lie beyond the scope of the current PROM framework. Determining which phenotypes can be modeled with by rough representations of mechanism and which phenotypes require more detailed, in-depth models can shed light into the complexity of the phenotypes themselves and inform future modeling efforts. Our logistic regression model enabled us to distinguish between TFs that are amenable to *MTB*PROM2.0 predictions and those that are not based on the network properties. This approach gave a network-based criterion with which to rank predictions that were most promising to follow-up in experiments. Although *in silico* predictions are easy to make in high throughput, it is often difficult to follow-up on more than a handful of predictions with in-depth experimental characterization. Therefore, having an accurate means to prioritize the predictions that are most likely to yield meaningful follow-up results will streamline the iterative process of investigation. Moreover, the ability to identify properties that make a TF hard to predict by the current regulatory-metabolic model also can direct method development towards improvements that can address the current limitations. In the case of using *MTB*PROM2.0 to predict TF overexpression growth phenotypes, the factors that could stratify the TFs suggest that being able to account for more complex regulatory mechanisms would improve predictive ability. Future method development to address combinatorial regulation and the effects of both activation and repression may help to improve overall predictive accuracy for regulatory-metabolic models.

## Methods

### Metabolic model growth phenotype simulations

Growth phenotype simulations were performed on the genome-scale metabolic models using the flux balance analysis tools in the COBRA Toolbox [28, 32]. Growth rates were generated by calculating the optimal value of the flux of the biomass generation reaction, which was set as the objective function in these simulations. Single gene deletion simulations calculated the growth rates with the reactions that map to each individual gene set to have flux rates of zero.

### Evaluating gene perturbation predictive performance

To evaluate the ability of the metabolic and regulatory-metabolic models to predict gene knockout growth defects, single gene deletion growth simulations for each of the models were compared to an experimental essentiality screening dataset generated by Griffin et al. [27]. The Griffin essentiality dataset screened for essential genes from transposon mutagenesis libraries by assessing the set of transposon insertions detected after growing the pooled transposon mutants for a period of time [27]. The authors summarized their findings with an essentiality confidence score for each gene that ranges from 0 to 1 that represents the probability that particular gene is non-essential (low confidence scores indicate that a gene is likely essential, and high confidence scores indicate that a gene is likely non-essential). We used the experimental data to separate the genes into two groups: those that have a defect when perturbed and those that do not. We considered genes with Griffin essentiality confidence scores of less than 0.1 to be highly confident essential, and we evaluated the ability of the models to successfully simulate growth rates that can distinguish these essential genes. For TF overexpression prediction validation, we compared growth predictions against experimental doubling time ratio data of strains of MTB with overexpression induced vs. uninduced (described in [26]).

For all gene perturbation predictions, performance was evaluated based on how well the prediction method could correctly separate the effects of the TF perturbations, where a true positive is a TF that is correctly predicted to cause a defect, and a true negative is a TF that is correctly predicted to cause no growth defect. Performance was summarized by precision $\left(\frac{TP}{TP+FP}\right)$, recall $\left(\frac{TP}{TP+FN}\right)\;$ and the Matthew’s correlation coefficient (MCC), a metric that reduces bias for uneven category sizes and reports values between -1 (low performance) and 1 (high performance) $\left(MCC\;=\;\frac{TP*TN-FP*FN}{\sqrt{\left(TP+FP\right)*\left(TP+FN\right)*\left(TN+FP\right)*(TN+FN)}}\right)$ [33]. In each metric, *TP* represented the ‘true positives’, *TN* represented the ‘true negatives,’ *FP* represented the ‘false positives,’ and *FN* represented the ‘false negatives.’

### Evaluating metabolic model carbon and nitrogen source utilization performance

To evaluate the ability for the metabolic models to predict growth under different environmental conditions, we simulated growth under different carbon and nitrogen sources using the COBRA Toolbox by adjusting the exchange reaction fluxes to allow input of the desired metabolite. Growth predictions on diverse carbon and nitrogen sources were compared to experimental growth condition data reported in [23]. We additionally included palmitate, oleate, carbon monoxide, and cholesterol as carbon sources that have been experimentally reported elsewhere [34–36]. In our evaluation of carbon and nitrogen utilization predictions, we considered only the metabolites that were represented in the genome-scale metabolic models. When possible, we fixed the exchange reaction fluxes of the metabolites that we were not adjusting to match the constituents in Sauton’s defined media [37], ensured that removal of the nitrogen or carbon source would result in no growth, and determined whether each metabolic model would grow in the presence of the different carbon and nitrogen sources. One of the metabolic models we tested could not grow with Sauton’s media-defined conditions. Therefore for this case, we set the exchange fluxes to match the components of Middlebrook 7H9 media [37]. When the carbon source was varied, the Sauton’s media nitrogen sources were used, and when the nitrogen source was varied, the Sauton’s media carbon source was used. In evaluating predictive performance, we calculated the MCC, precision, and recall by designating true positives as the number of metabolites correctly predicted to allow growth, and true negatives as the number of metabolites correctly predicted to not allow growth.

### Modifying PROM to predict transcription factor overexpression

The original PROM approach to estimating the influence of a regulatory interaction in the event of a TF knockout was to calculate from a gene expression dataset the conditional probability that a target gene is ‘ON’ in the absence of the transcription factor:

$$\text{P}(\text{Gene }=\;ON\;|\text{ TF }=\;OFF)\;\approx \frac{\text{Number of samples with Gene = }ON\text{ AND TF=}OFF}{\text{Number of samples with TF=}OFF}$$ (1)

The expression threshold that delineated between the ‘ON’ and ‘OFF’ states could be either set externally or calculated to be a desired quantile from the input expression data. These conditional probabilities were then used to constrain the maximal fluxes of the reactions (above which a penalty was incurred) catalyzed by the gene products in the metabolic model.

To predict the regulatory effects of TF overexpression, we adapted the conditional probability calculation to estimate the probability that a target gene is ‘ON’ when the expression of the transcription factor is above a threshold value denoted ‘OVEREXPRESS’:

$$\text{P}(\text{Gene }=\;ON\;|\text{ TF }=\;OVEREXPRESS)\;\approx \frac{\text{Number of samples with Gene = }ON\text{ AND TF=}OVEREXPRESS}{\text{Number of samples with TF=}OVEREXPRESS}$$ (2)

This formulation required setting two expression thresholds: one that delineated between ‘ON’ and ‘OFF’ states, and another that delineated between ‘ON’ and ‘OVEREXPRESS,’ which transformed the numerical expression values into the three expression states. This formulation is compatible with any gene expression datasets, not just profiles of TF overexpression. It is important to note also that this model only takes into account the effect of genes that are repressed by transcription factors. The effects of target genes that were activated by the overexpression of a transcription factor fell outside the scope of this model. The code for simulating TF knockouts and overexpression is available in S2 File, with necessary input files provided in S3 File.

### Sampling-based inference of regulatory interaction influences in *MTB*PROM2.0

Given that the calculation of the conditional probabilities is dependent on the gene expression dataset being used, we used a sampling approach to estimate the uncertainty of the conditional probabilities. We used microarray data profiling individual TF overexpression perturbations [26] to estimate conditional probabilities. This approach is particularly suited for our sampling approach because it includes at least three biological replicates that measure the overexpression of each TF. Therefore, we sampled the dataset 500 times by randomly selecting for each TF three replicates measuring the overexpression and calculated the conditional probability from the resulting assembled data. The MATLAB code for simulating TF knockouts and overexpression is available in S2 File.

### Generating condition-specific iMAT models

We compared the ability of *MTB*PROM2.0 to predict transcription factor overexpression phenotypes using condition-specific metabolic models generated using the iMAT algorithm [9, 10]. We used the TF overexpression microarray dataset to generate iMAT models to simulate the metabolic state of each TF overexpression condition [26]. For each TF, the overexpression microarray data were binarized such that a gene was designated ‘ON’ if it had a positive fold change value in at least 75% of the samples, and was designated as ‘OFF’ otherwise. These binarized data were then used to generate a condition-specific iMAT model and reaction flux profile from the COBRA Toolbox implementation of iMAT [9, 10, 28]. The growth ratio of each TF overexpression condition was calculated by taking the ratio of growth rate derived from the iMAT flux profile and the growth rate simulated from the wild-type model, iSM810.

To simulate drug-specific metabolic models, we applied iMAT to constrain iSM810 based on the transcriptional profiles of MTB response to different drugs measured by arrays provided by TIGR under the NIAID contract N01-AI-15447. To binarize the expression data, genes with log_2_ fold change < -1 upon exposure to drug were designated as ‘OFF.’ Drug-specific iMAT models were generated and used to simulate TF perturbations by integrating with the *MTB*PROM2.0 regulatory framework.

### Estimating the confidence of *MTB*PROM2.0 predictions

To estimate a confidence score for how likely the *MTB*PROM2.0 prediction of a particular TF was likely to be correct, we trained a logistic regression model with the R package “stats” [38] using different properties associated with the regulatory network architecture and the target genes. Logistic regression is a supervised machine-learning model [39] designed to learn the probability that a particular response *Y* belongs to a particular category given the state of input predictor features (*X*_*1*_,…, *X*_p_) based on the following relation:

$$P\left(Y=category\text{|}X_{1}\ldots X_{p}\right)=\frac{\text{exp}\left(\beta_{0}+\beta_{1}X_{1}+\ldots +\beta_{p}X_{p}\right)}{1+\text{exp}\left(\beta_{0}+\beta_{1}X_{1}+\ldots +\beta_{p}X_{p}\right)}$$ (3)

In this case, the category we predicted was whether the *MTB*PROM2.0 prediction for a TF overexpression growth defect would be ‘TRUE’ or ‘FALSE,’ where ‘TRUE’ indicates either a true positive or a true negative prediction, and ‘FALSE’ indicates either a false positive or a false negative prediction. The predictor features we used to train the initial model were the number of metabolic target genes, the fraction of target genes that were highly confident essential based on Griffin data, and the average number of additional regulators that the target genes had. To refine the logistic regression model, we retrained the model using only the features that were evaluated to be significant by the z-statistic [39]. To test the predictive performance of the refined logistic regression model, we performed ten-fold cross-validation using the R package “cvTools” [40], and calculated the average MCC across the iterations.

### Experimental culturing conditions

The MTB strain H37Rv and the strain containing an ATc-inducible expression vector for the TF gene of interest, *whiB4*, were cultured as described previously [26, 30]. Briefly, the strains were grown in Middlebrook 7H9 with the ADC supplement (Difco), 0.05% Tween80 at 37°C with constant agitation. The strain containing the ATc- inducible expression vector was grown with the addition of 50 μg/mL hygromycin B to maintain the plasmid. All experiments were performed under aerobic conditions and growth was monitored by OD600. At an OD600 of approximately 0.1, the cultures were supplemented with either the drug of interest in DMSO solution or DMSO as no-drug control. Concurrently, expression of *whiB4* was induced using an ATc concentration of 100 ng/mL culture. Growth was monitored via OD600 for a period of 14 days post drug supplementation and induction of *whiB4* overexpression. MTB metabolism was measured by the extent of conversion of the oxidation-reduction dye, Alamar Blue [31]. Ten percent by volume of Alamar Blue reagent was added to wells, and, after 2 and 24 hours of incubation, results were measured by fluorescence (excitation 544nm, emission 590 nm). Percentage of reduction of AlamarBlue was calculated according to the manufacturer's instructions.

## Supporting Information

S1 Text Supplemental information document. (PDF)

S1 Table Model contents of *Mycobacterium tuberculosis* iSM810. (XLSX)

S2 Table Condition-specific TF knockout predictions for *MTB*PROM2.0. (XLSX)

S3 Table Condition-specific TF overexpression predictions for *MTB*PROM2.0. (XLSX)

S4 Table Drug-TF perturbation combination synergy predictions for *MTB*PROM2.0. (XLS)

S1 File SBML and MATLAB versions of iSM810. The.xml version has exchange fluxes set to match Middlebrook 7H9 growing conditions. MATLAB files contains a model version with exchange fluxes set to match Middlebrook 7H9 growing conditions and a model version with all exchange fluxes set to 0. File also contains an array detailing which reactions are exchange reactions. Can be imported into the COBRA Toolbox.
(ZIP)

S2 File *MTB*PROM2.0 simulation codes. File contains codes to run knockout and overexpression simulations, where the conditional probability is calculated from the entire input expression dataset (promv2.m, and promv2TFOE.m, respectively), as well as code for simulating where the conditional probability is calculated with sampling (promSampling.m). Code for simulating TF-metabolic gene double knockouts is also provided (PROMdoubleKO.m). Additionally, code describing how to generate the carbon and nitrogen source models is included (Metabolite-condition-specific-ModelCode.m).
(ZIP)

S3 File Input files for *MTB*PROM2.0 simulations. Code in S3 File includes instructions on how to use these input files.
(ZIP)
