# Supplementary material for: Integrated Modeling of Gene Regulatory and Metabolic Networks in Mycobacterium tuberculosis
Source: PLoS Comput Biol. 2015 Nov 30;11(11):e1004543. doi: 10.1371/journal.pcbi.1004543 (PMC4664399; doi:10.1371/journal.pcbi.1004543)
Supplement: S1 Text — (PDF) [file pcbi.1004543.s001.pdf]

Supplemental Information

Table of Contents

Visualization of the Updated Regulatory Component of *MTB*PROM2.0 1
Updating the Metabolic Model of MTB 2
Evaluating the Predictive Ability of the iSM810 3
TF knockout essentiality predictions 8
Estimating the confidence of PROM TF overexpression phenotype predictions 9
TF-drug synergy predictions 10
References 11

Visualization of the Updated Regulatory Component of *MTB*PROM2.0

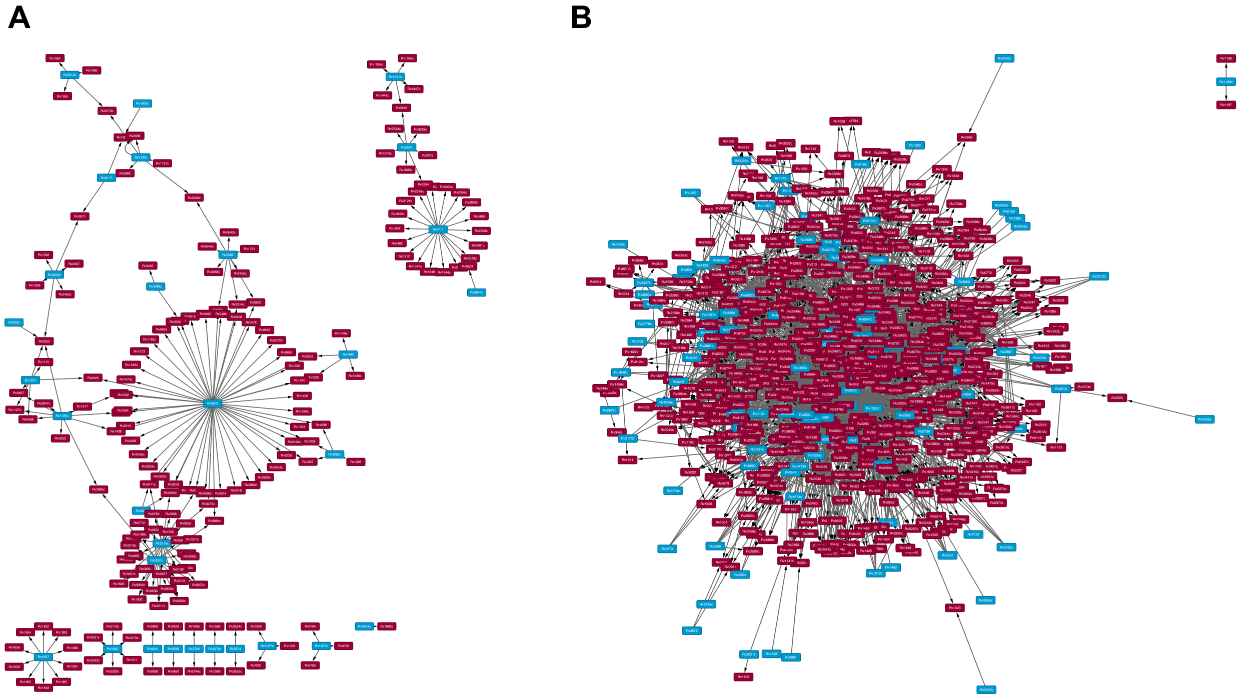

Figure S1. Visual comparison of the regulatory component of the MTB regulatory-metabolic models. (A) Regulatory component from *MTB*PROM1.0, based on the Balazsi 2008 network [1]. (B) Regulatory component from *MTB*PROM2.0 based on the Minch 2015 network [2]. Transcription factors are nodes colored blue, and metabolic genes are nodes colored red.

Updating the Metabolic Model of MTB

The metabolic component of *MTB*PROM2.0 is a refined genome-scale metabolic model, called *Mycobacterium tuberculosis* iSM810, which contains updated biochemical reaction information extracted from the multiple existing genome-scale reconstructions of MTB metabolism that are described below. The original integrated model, *MTB*PROM1.0, incorporated the genome-scale metabolic model iNJ661, which was reconstructed based on the genome annotation of MTB strain H37Rv in 2007 [3]. The model iNJ661 contains 1025 reactions based on evidence from homology and experiments from literature that have been mapped to 661 genes. Four other genome-scale metabolic network have been reconstructed for MTB (GSMN-TB by [4], GSMN-TB1.1 by [5], iNJ661m by [6], and HQ-Mtb by [7] (see Table S1 for summary of network properties).

Table S1: Summary of existing genome-scale metabolic models for in vitro MTB.

Genome-scale metabolic model	Reference	Number of Genes	Number of Reactions	Number of Metabolites
**GSMN-TB**	[4]	726	856	645
**GSMN-TB 1.1**	[5]	759	876	667
**iNJ661**	[3]	661	1025	826
**iNJ661m**	[6]	663	1049	838
**HQMtb**	[7]	686	607	734

To assess their utility in contributing to an updated metabolic model, we compared the existing metabolic models by two criteria: 1) model contents and 2) their respective abilities to simulate metabolic phenotypes under varying genetic and environmental conditions. GSMN-TB1.1 captures the effect of the greatest number of metabolic genes (759 genes), which enables the broadest scope of genetic perturbation simulations. The model iNJ661m has the greatest number of total reactions described (1049 reactions), although GSMN-TB1.1 contains more reactions that are based on literature-derived, experimental biochemical evidence (279 reactions in GSMN-TB1.1 vs. 204 reactions in iNJ661m).

In evaluating the respective abilities to simulate metabolic phenotypes under varying genetic and environmental conditions, we compared GSMN-TB, GSMN-TB1.1, iNJ661, and iNJ661m (the metabolic reconstruction HQMtb does not include explicit gene-protein-reaction-associations for all reactions or a biomass objective function, which precludes metabolic phenotype simulations by flux balance analysis).

To compare the ability of the models to correctly simulate growth under different environmental conditions, we simulated growth of the four models with different input carbon and nitrogen sources. We compared these growth simulations with experimental carbon and nitrogen source growth data from [8-10]. We considered only carbon and nitrogen sources that already existed as metabolites in the models, which total 51 different carbon sources and 40 different nitrogen sources. Of the carbon sources considered, MTB has been experimentally shown to be able to grow with 29 of the metabolites as the sole carbon source. Of the nitrogen sources, MTB has been shown to grow with 14 of the metabolites as the sole nitrogen source.

Tables S2-S5 list detailed carbon and nitrogen source growth prediction and experiment information. GSMN-TB1.1 and GSMN-TB can predict the growth rate of MTB under the broadest range of simulated media conditions. GSMN-TB1.1 correctly predicts an ability to grow with 23 compounds as the sole carbon source and 12 compounds as the sole nitrogen source (69% sensitivity, 55% specificity for the carbon sources; 86% sensitivity, 85% specificity for the nitrogen sources), whereas GSMN-TB can correctly predicts an ability to grow with 20 compounds as the sole carbon source and 12 compounds as the sole nitrogen source (79% sensitivity, 45% specificity for the carbon sources; 86% sensitivity, 77% specificity for the nitrogen sources). In contrast, iNJ661 and iNJ661m correctly predict an ability to grow only on glycerol as the sole carbon source, and 10 compounds as the sole nitrogen source (3% sensitivity on carbon sources; 71% sensitivity, 88% specificity for the nitrogen sources).

To each model's ability to predict the effect of genetic perturbations, we compared metabolic consequences of simulating single gene deletions based on the models GSMN-TB, GSMN-TB1.1, and iNJ661, under media conditions stipulated by Griffin et al., and we compared the simulation results to corresponding experimental gene essentiality data [11] (we did not include iNJ661m in these calculations because the unperturbed model was not able to simulate growth under the Griffin media conditions). Figure S2 shows the Matthews Correlation Coefficients (MCCs) evaluating the performance of single gene deletion simulations based on the different metabolic models. The highest predictive accuracy was achieved with GSMN-TB1.1 (MCC = 0.51) and GSMN-TB (MCC = 0.51), with iNJ661 achieving an MCC = 0.27.

Given that GSMN-TB1.1 can predict growth phenotypes under the broadest range of environmental conditions and genetic perturbations with the greatest accuracy, this model had the most appealing properties for simulating growth phenotypes in this study.

Although GSMN-TB1.1 has favorable modeling properties, the models iNJ661 and HQMtb contain information on reactions known to be catalyzed by MTB that are not captured in GSMN-TB1.1. To consolidate the knowledge base of metabolism represented by a genome-scale metabolic model, we updated GSMN-TB1.1 by integrating the gene-associated reactions with literature evidence in iNJ661 and HQ-Mtb that were absent from GSMN-TB1.1. The resulting updated metabolic model, *Mycobacterium tuberculosis* iSM810, expands the consolidated knowledgebase of MTB metabolism represented by the model, with 51 new genes and 57 new literature-associated reactions spanning a broad range of metabolic functions (see Table 3 for full list of reactions added to the model). The updated model has added reactions needed for the metabolism of cholesterol, glycogen, and carbon monoxide.

Evaluating the Predictive Ability of the iSM810

To evaluate the ability of *M. tuberculosis* iSM810 to predict the growth effects of genetic perturbations, we compared single gene deletion growth predictions to the Griffin essentiality data [11] (see main text Methods for details). We found that iSM810 predicted gene essentiality with MCC = 0.52, which is comparable to the performance of GSMN-TB and GSMN-TB1.1 (see Figure S2).

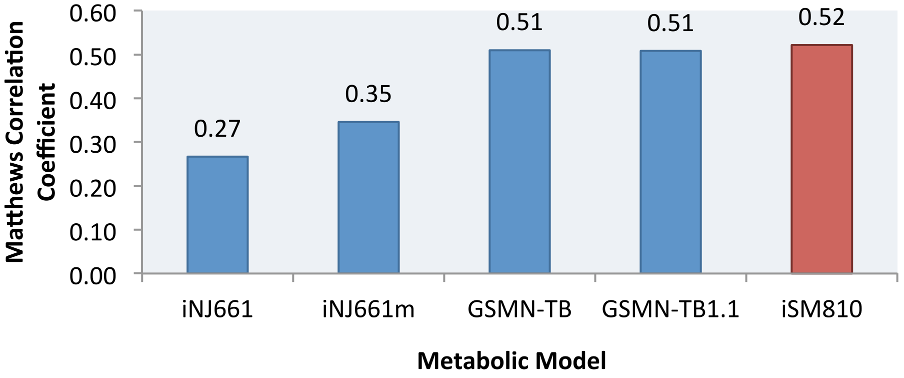

Figure S2. Comparing performance of predicting metabolic gene essentiality. Performance is evaluated by the Matthews Correlation Coefficient. iSM810 (red bar) is comparable to GSMN-TB and GSMN-TB1.1, and GSMN-TB and GSMN-TB1.1 perform better than iN661m and iNJ661.

We also evaluated the ability for iSM810 to simulate growth under a broad range of media conditions. We compared experimentally derived media-specific growth phenotypes with growth phenotypes predicted by the metabolic model (see main text Methods for details). Tables S2-S5 list the growth abilities that were predicted by iSM810 and Figure S3 compares its predictive performance with the other metabolic models. The updated model, iSM810, correctly predicts an ability to grow with 24 compounds as the sole carbon source and 12 compounds as the sole nitrogen source (83% sensitivity, 55% specificity for the carbon sources; 86% sensitivity, 81% specificity for the nitrogen sources). Overall, iSM810 correctly predicts growth in a greater number of carbon and nitrogen sources than the other models.

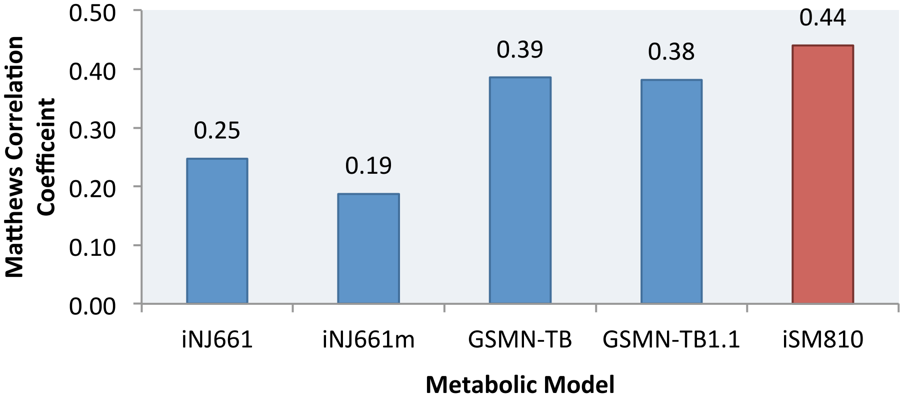

Figure S3. Comparison of ability to predict growth in different carbon and nitrogen sources. Performance is evaluated by the Matthews Correlation Coefficient (see Methods for description) calculated by combining the carbon and nitrogen source predictions together (i.e. considering all 91 metabolite predictions). iSM810 improves upon GSMN-TB and GSMN-TB1.1, and GSMN-TB and GSMN-TB1.1 perform better than iN661m and iNJ661.

Table S2. Summary of Growth Predictions for Metabolites that Can Be Used as Sole Carbon Source. '0' indicates no growth, and '1' indicates growth either measured experimentally (column 2) or as predicted by the metabolic models (column 3-6). Note that iNJ661 and iNJ661m yielded the same growth predictions.

Carbon sources	Experiment	iSM810	GMSN-TB1.1	GSMN-TB	iNJ661/iNJ661m
2-Oxoglutarate	1	1	1	1	0
Acetate	1	1	1	1	0
Acetoacetic Acid	1	0	0	0	0
Caproic Acid (hexanoate)	1	1	1	1	0
Cholesterol	1	1	0	0	0
Citrate	1	1	1	1	0
D-Glucose	1	1	1	1	0
D-Mannose	1	1	1	1	0
D-Trehalose	1	1	1	1	0
Glycerol	1	1	1	1	1
Glycine	1	1	1	0	0
L-Alanine	1	1	1	1	0
L-Arginine	1	0	0	0	0
L-Asparagine	1	1	1	0	0
L-Aspartic Acid	1	1	1	1	0
L-Glutamate	1	1	1	1	0
L-Isoleucine	1	1	1	1	0
L-Lactate	1	1	1	1	0
L-Malic Acid	1	1	1	1	0
L-Proline	1	1	1	1	0
L-Serine	1	1	1	0	0
Oleate (9-Octadecenoate)	1	1	1	1	0
Palmitate (Hexadecanoate)	1	1	1	1	0
Propanoate	1	1	1	1	0
Pyruvate	1	1	1	1	0
Succinate	1	1	1	1	0
Caprylate (Octanoate)	1	0	0	0	0
Mycolic Acid	1	0	0	0	0
Carbon Monoxide	1	0	0	0	0

Table S3. Summary of Growth Predictions for Metabolites that Cannot Be Used as Sole Carbon Source. '0' indicates no growth, and '1' indicates growth either measured experimentally (column 2) or as predicted by the metabolic models (column 3-6). Note that iNJ661 and iNJ661m yielded the same growth predictions.

Carbon sources	Experiment	iSM810	GMSN-TB1.1	GSMN-TB	iNJ661/iNJ661m
2-Deoxyadenosine	0	0	1	0	0
Adenosine	0	0	1	0	0
D-Cellobiose	0	0	0	1	0
D-Fructose	0	1	1	1	0
D-Galactose	0	1	1	1	0
D-Glucose-1-phosphate	0	0	1	0	0
D-Ribose	0	1	1	1	0
D,L -α-Glycerol phosphate	0	1	0	1	0
Formic Acid	0	0	0	0	0
Glycolic Acid	0	1	1	1	0
Inosine	0	0	1	0	0
L-Histidine	0	0	0	0	0
L-Leucine	0	0	0	0	0
L-Lysine	0	0	0	0	0
L-Methionine	0	0	0	0	0
L-Ornithine	0	1	0	0	0
L-Phenylalanine	0	0	0	0	0
L-Threonine	0	1	1	1	0
L-Valine	0	1	1	1	0
Maltose	0	1	1	1	0
Sucrose	0	1	1	1	0
Uridine	0	0	0	0	0

Table S4. Summary of Growth Predictions for Metabolites that Can Be Used as Sole Nitrogen Source. '0' indicates no growth, and '1' indicates growth either measured experimentally (column 2) or as predicted by the metabolic models (column 3-7).

Nitrogen sources	Experiment	iSM810	GMSN-TB1.1	GSMN-TB	iNJ661	iNJ661m
Ammonia	1	1	1	1	1	1
Glycine	1	1	1	1	1	1
L-Alanine	1	1	1	1	1	1
L-Arginine	1	0	1	0	1	1
L-Asparagine	1	1	1	1	1	1
L-Aspartic Acid	1	1	1	1	1	1
L-Cysteine	1	0	0	0	0	0
L-Glutamic Acid	1	1	1	1	1	1
L-Glutamine	1	1	1	1	0	0
L-Isoleucine	1	1	1	1	0	0
L-Ornithine	1	1	0	1	1	1
L-Proline	1	1	1	1	1	1
L-Serine	1	1	1	1	1	1
L-Valine	1	1	1	1	0	0

Table S5. Summary of Growth Predictions for Metabolites that Cannot Be Used as Sole Nitrogen Source. '0' indicates no growth, and '1' indicates growth either measured experimentally (column 2) or as predicted by the metabolic models (column 3-7).

Nitrogen sources	Experiment	iSM810	GMSN-TB1.1	GSMN-TB	iNJ661	iNJ661m
Adenine	0	0	1	0	0	0
Adenosine	0	0	1	0	0	0
D-Alanine	0	1	1	1	1	1
D-Glutamic Acid	0	0	0	0	0	0
Guanine	0	0	0	0	0	0
Guanosine	0	0	0	0	0	0
Inosine	0	0	0	0	0	0
L-Citrulline	0	0	0	0	0	0
L-Histidine	0	0	0	0	0	0
L-Homoserine	0	0	1	0	0	0
L-Leucine	0	0	0	0	0	0
L-Lysine	0	0	0	0	0	0
L-Methionine	0	0	0	0	0	0
L-Phenylalanine	0	0	0	0	1	1
L-Threonine	0	1	1	1	0	0
L-Tryptophan	0	0	0	0	1	1
L-Tyrosine	0	0	0	0	0	0
N-Acetyl-D,L Glutamic Acid	0	0	0	0	0	0
Nitrate	0	0	0	0	0	1
Nitrite	0	1	0	0	0	1
Uracil	0	0	0	0	0	0
Urea	0	1	1	1	0	0
Uridine	0	0	0	0	0	0
Xanthine	0	0	0	0	0	0
Xanthosine	0	0	0	0	0	0
γ-Amino-N Butyric Acid	0	1	0	1	0	0

TF knockout essentiality predictions

The performance of *MTB*PROM1.0 and *MTB*PROM2.0 at identifying essential gene knockouts is dependent upon the values chosen for multiple parameters, including (1) the cutoff threshold of the Griffin essentiality score used to delineate between essential and non-essential genes in the experimental validation dataset and (2) the cutoff threshold of the predicted relative growth rates used to designate essential vs. non-essential genes in the PROM models. To assess the degree to which these parameters affected the performance of the models, we generated a series Receiver Operating Curves (ROCs) using the R package ‘pROC’ [12] by adjusting both of the aforementioned parameters (see Figure S4). We find that *MTB*PROM2.0 displays overall improved performance compared to *MTB*PROM1.0 across different selections of parameters (evaluated by AUC of the ROC curves, see Figure S5).

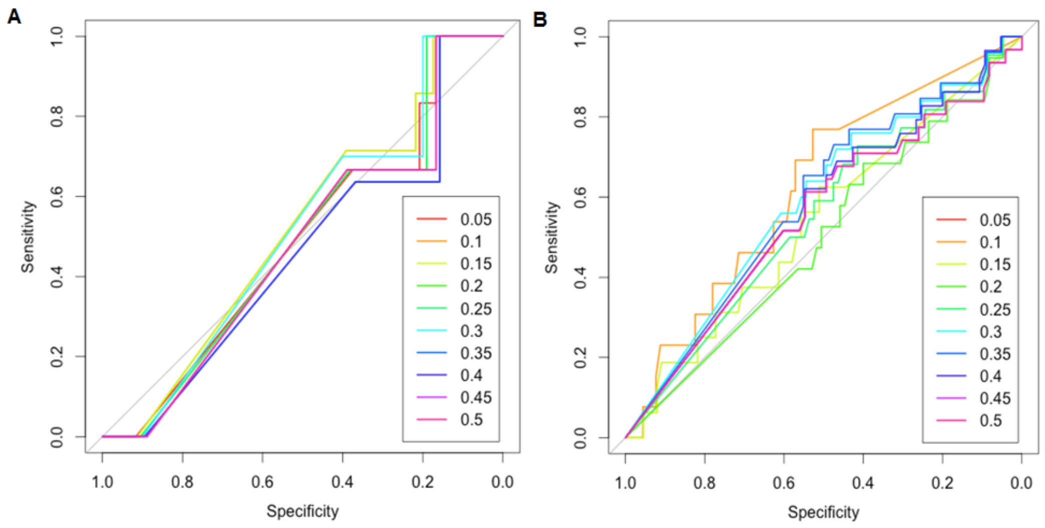

Figure S4. Receiver Operating Curves showing performance of the regulatory-metabolic models at predicting gene essentiality. (A) Performance of *MTB*PROM1.0. (B) Performance of *MTB*PROM2.0. The different colored lines represent different Griffin score threshold cutoffs for defining essentiality in the experimental data, ranging from 0.05 to 0.5. Each ROC curve was generated by varying the cutoff threshold of the predicted relative growth rates used to define the model-based essentiality assignments.

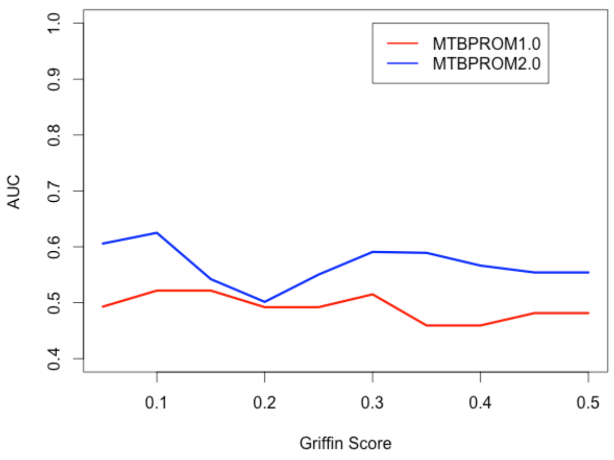

Figure S5. Areas under curves of ROC curves as a function of the Griffin Score cutoff threshold for designating gene essentiality. The AUCs were calculated from the curves generated in Figure S4. The AUCs of *MTB*PROM2.0 remained higher than those evaluating the performance of *MTB*PROM1.0.

Estimating the confidence of PROM TF overexpression phenotype predictions

To estimate the confidence of the *MTB*PROM2.0 TF overexpression predictions, we applied a logistic regression model to associate the prediction outcomes with model features. Table S6 shows the logistic regression parameters tested. Of the variables tested, two features were significantly associated with correct *MTB*PROM2.0 predictions. The negative association between the average number of regulatory interactions that the essential target genes have and the likelihood of a true *MTB*PROM2.0 prediction indicates that *MTB*PROM2.0 is more likely to predict correctly for TFs linked to metabolic genes that have simple regulatory architectures (i.e., those that have few influences from TFs other than the one being perturbed). The positive association between the likelihood that a *MTB*PROM2.0 prediction is true and whether the *MTB*PROM2.0 prediction matches the iMAT prediction suggests that performance depends on whether the influence of a TF upon its targets can be successfully estimated from the gene expression data. Increased disagreement between iMAT and *MTB*PROM2.0 predictions suggests uncertainty in the estimates of the TF influence on its targets, which would confound the assumptions made by the *MTB*PROM2.0 TF influence estimation approach. Using a logistic regression model constructed with these two significant features, we identified 46 TFs with network properties that yield higher confidence *MTB*PROM2.0 predictions. The TFs selected by the logistic regression model were statistically enriched for correct *MTB*PROM2.0 predictions (p < 10^-4, hypergeometric test). The prediction results of these high confidence TFs were reported in the main text.

Table S6. Logistic regression feature variables. The following network properties were applied to a logistic regression model to estimate the confidence of each TF overexpression growth prediction made by *MTB*PROM2.0. Features with starred p-values were found to be significantly associated with ability to distinguish between TFs predicted correctly vs. incorrectly by *MTB*PROM2.0 when a logistic regression model containing solely these variables was tested.

Coefficient	Estimate	Standard Error	Z Value	P(>|z|)
iMAT agree PROM	2.0	0.57	3.56	0.0004*
Average # TFs for essential targets	-0.44	0.19	-2.4	0.02*
# Metabolic targets	0.12	0.06	2.0	0.04
PROM growth variance	-190	114	-1.7	0.08
# Essential metabolic targets	-0.19	0.16	-1.1	0.26
# Combinatorial TFs for essential targets	-0.17	0.21	-0.84	0.40
Fraction essential targets	0.45	0.99	0.46	0.65

TF-drug synergy predictions

A major challenge in developing new therapies for TB is finding effective combinations of drug treatments. Integrating *MTB*PROM2.0 with condition-specific metabolic models enables predictions of the effect of combinatorial perturbations on the growth consequences of MTB. An application relevant to therapeutic development efforts is to predict novel drug targets that will synergize with the activity of antibacterial agents. Using the transcriptional profiles of MTB response to different drugs (described in the main text), we generated drug-specific metabolic models based on iSM810 using the iMAT algorithm. To binarize the expression data, genes with $\log_2$ fold change < -1 upon exposure to drug were designated as 'OFF.' The iMAT algorithm applies these binarized data to constrain iSM810 by maximizing the fit of reaction flux state to catalyzing enzyme expression state [13, 14]. The TF perturbations (overexpression and knockout) are simulated in the context of these drug-specific models using the *MTB*PROM2.0 framework.

Figure S6 shows heatmaps summarizing the predictions of relative growth rate of each drug-TF perturbation combination, relative to the growth rate predicted for each drug in absence of TF perturbation and growth rate predicted for each TF perturbation in the absence of each drug (Panel A shows knockouts, Panel B shows overexpression perturbations). The rows represent the TF perturbations, and the columns represent the drugs. Black bars spanning the rows and columns represent TF perturbations and drugs that are predicted to be lethal to growth irrespective of secondary perturbations (See S4 Table for the full results).

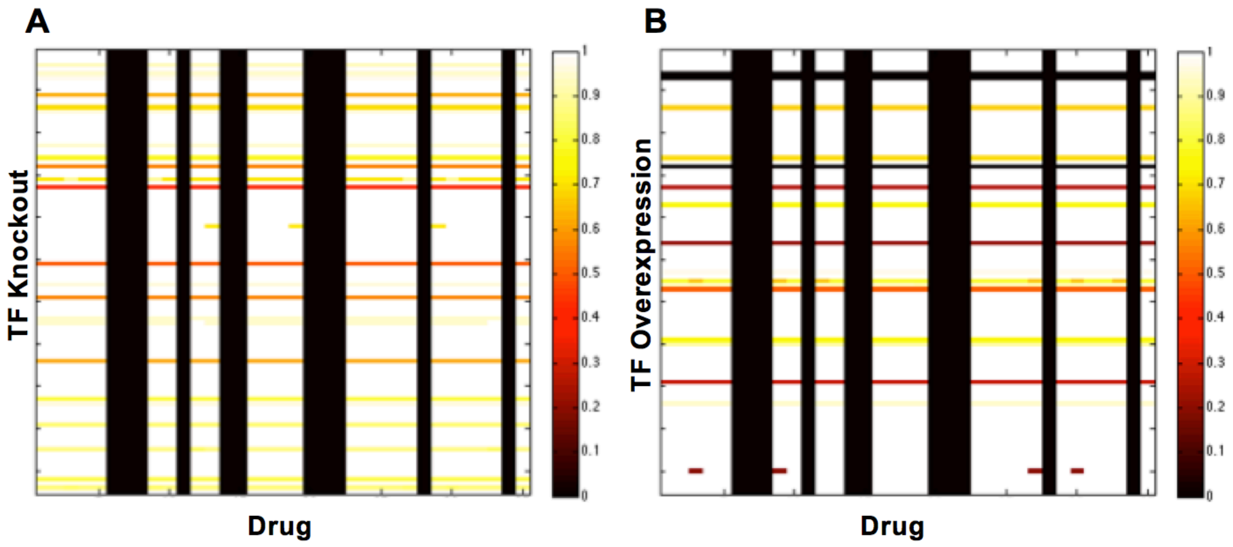

Figure S6. Heatmaps of relative growth rate simulated from TF-drug combinatorial perturbations. (A) TF knockout-drug synergy predictions. (B) TF overexpression-drug synergy perturbations. Darker colors indicate greater growth defect. The rows represent the TF perturbations, and the columns represent the drugs. Black bars spanning the rows and columns represent TF perturbations and drugs that are predicted to be lethal to growth irrespective of secondary perturbations.

References

1. Balazsi G, Heath AP, Shi L, Gennaro ML. The temporal response of the Mycobacterium tuberculosis gene regulatory network during growth arrest. Mol Syst Biol. 2008;4:225. Epub 2008/11/06. doi: msb200863 [pii]
10.1038/msb.2008.63. PubMed PMID: 18985025.
2. Minch KJ, Rustad TR, Peterson EJ, Winkler J, Reiss DJ, Ma S, et al. The DNA-binding network of Mycobacterium tuberculosis. Nat Commun. 2015;6:5829. doi: 10.1038/ncomms6829. PubMed PMID: 25581030; PubMed Central PMCID: PMC4301838.
3. Jamshidi N, Palsson BO. Investigating the metabolic capabilities of Mycobacterium tuberculosis H37Rv using the in silico strain iNJ661 and proposing alternative drug targets. BMC Syst Biol. 2007;1:26. Epub 2007/06/09. doi: 1752-0509-1-26 [pii]
10.1186/1752-0509-1-26. PubMed PMID: 17555602.
4. Beste DJ, Hooper T, Stewart G, Bonde B, Avignone-Rossa C, Bushell ME, et al. GSMN-TB: a web-based genome-scale network model of Mycobacterium tuberculosis metabolism. Genome Biol. 2007;8(5):R89. Epub 2007/05/25. doi: gb-2007-8-5-r89 [pii]
10.1186/gb-2007-8-5-r89. PubMed PMID: 17521419.
5. Lofthouse EK, Wheeler PR, Beste DJ, Khatri BL, Wu H, Mendum TA, et al. Systems-based approaches to probing metabolic variation within the Mycobacterium tuberculosis complex. PLoS One. 2013;8(9):e75913. Epub 2013/10/08. doi: 10.1371/journal.pone.0075913
PONE-D-13-19042 [pii]. PubMed PMID: 24098743.
6. Fang X, Wallqvist A, Reifman J. Development and analysis of an in vivo-compatible metabolic network of Mycobacterium tuberculosis. BMC Syst Biol. 2011;4:160. Epub 2010/11/26. doi: 1752-0509-4-160 [pii]
10.1186/1752-0509-4-160. PubMed PMID: 21092312.

7. Kalapanulak S. High quality genome-scale metabolic network reconstruction of Mycobacterium tuberculosis and comparison with human metabolic network: application for drug targets identification. Edinburgh: University of Edinburgh; 2009.
8. Dunphy KY, Senaratne RH, Masuzawa M, Kendall LV, Riley LW. Attenuation of Mycobacterium tuberculosis functionally disrupted in a fatty acyl-coenzyme A synthetase gene fadD5. J Infect Dis. 2010;201(8):1232-9. Epub 2010/03/11. doi: 10.1086/651452. PubMed PMID: 20214478.
9. Park SW, Hwang EH, Park H, Kim JA, Heo J, Lee KH, et al. Growth of mycobacteria on carbon monoxide and methanol. J Bacteriol. 2003;185(1):142-7. Epub 2002/12/18. PubMed PMID: 12486050.
10. Yang X, Dubnau E, Smith I, Sampson NS. Rv1106c from Mycobacterium tuberculosis is a 3beta-hydroxysteroid dehydrogenase. Biochemistry. 2007;46(31):9058-67. Epub 2007/07/17. doi: 10.1021/bi700688x. PubMed PMID: 17630785.
11. Griffin JE, Gawronski JD, Dejesus MA, Ioerger TR, Akerley BJ, Sassetti CM. High-resolution phenotypic profiling defines genes essential for mycobacterial growth and cholesterol catabolism. PLoS Pathog. 2011;7(9):e1002251. Epub 2011/10/08. doi: 10.1371/journal.ppat.1002251
PPATHOGENS-D-11-00689 [pii]. PubMed PMID: 21980284.
12. Robin X, Turck N, Hainard A, Tiberti N, Lisacek F, Sanchez JC, et al. pROC: an open-source package for R and S+ to analyze and compare ROC curves. BMC Bioinformatics. 2011;12:77. Epub 2011/03/19. doi: 1471-2105-12-77 [pii]
10.1186/1471-2105-12-77. PubMed PMID: 21414208.
13. Zur H, Ruppin E, Shlomi T. iMAT: an integrative metabolic analysis tool. Bioinformatics. 2010;26(24):3140-2. Epub 2010/11/18. doi: btq602 [pii]
10.1093/bioinformatics/btq602. PubMed PMID: 21081510.
14. Shlomi T, Cabili MN, Herrgard MJ, Palsson BO, Ruppin E. Network-based prediction of human tissue-specific metabolism. Nat Biotechnol. 2008;26(9):1003-10. Epub 2008/08/20. doi: nbt.1487 [pii]
10.1038/nbt.1487. PubMed PMID: 18711341.
